# Supplementary figures and images for: Validation and evaluation of a tablet-based dietary record app for adults aged 70 and above
Source: PLoS One. 2025 Nov 25;20(11):e0337534. doi: 10.1371/journal.pone.0337534 (PMC12646446; doi:10.1371/journal.pone.0337534)

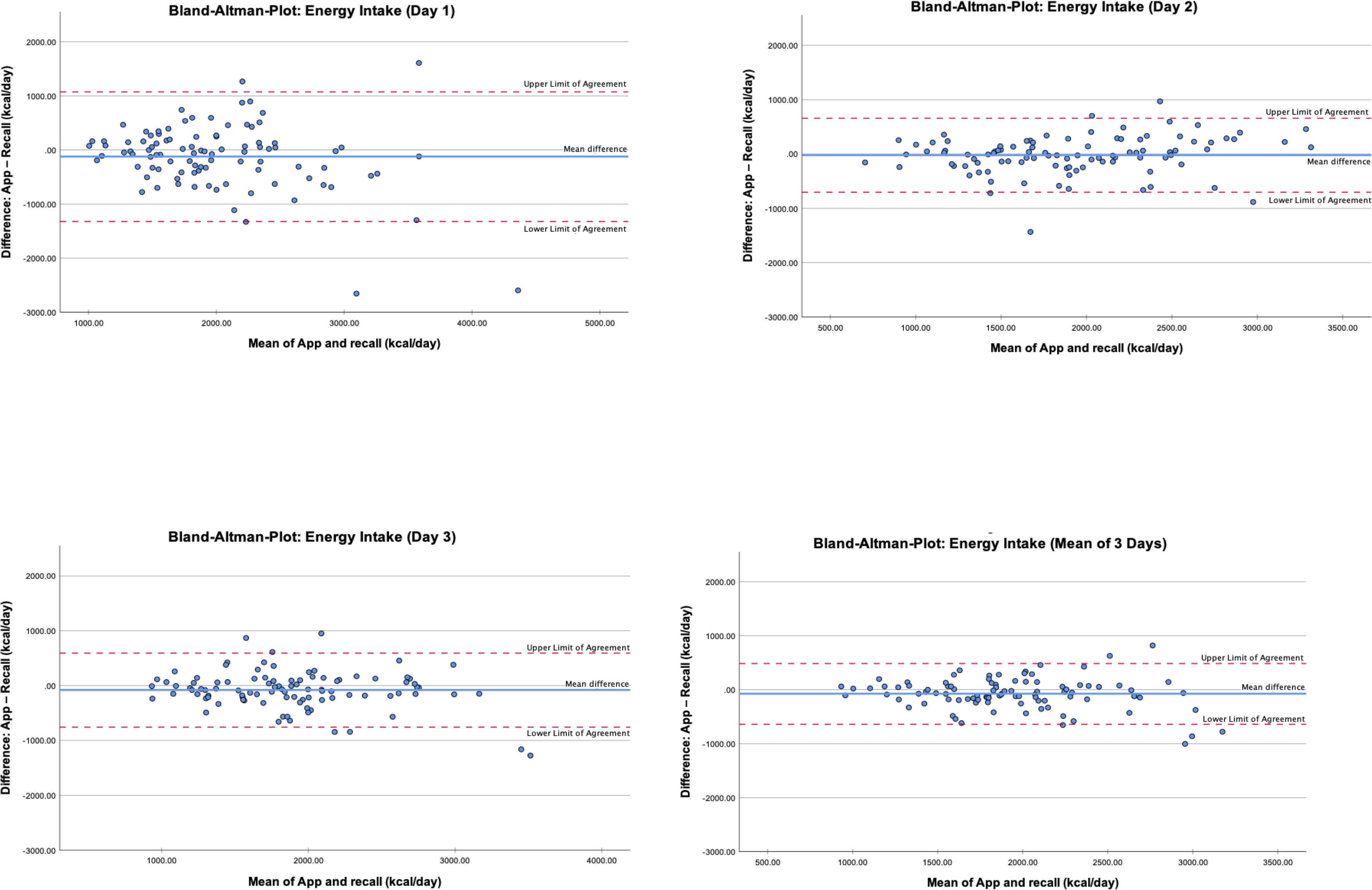

Supplement: S1 Fig — (TIF) [file pone.0337534.s001.tif]

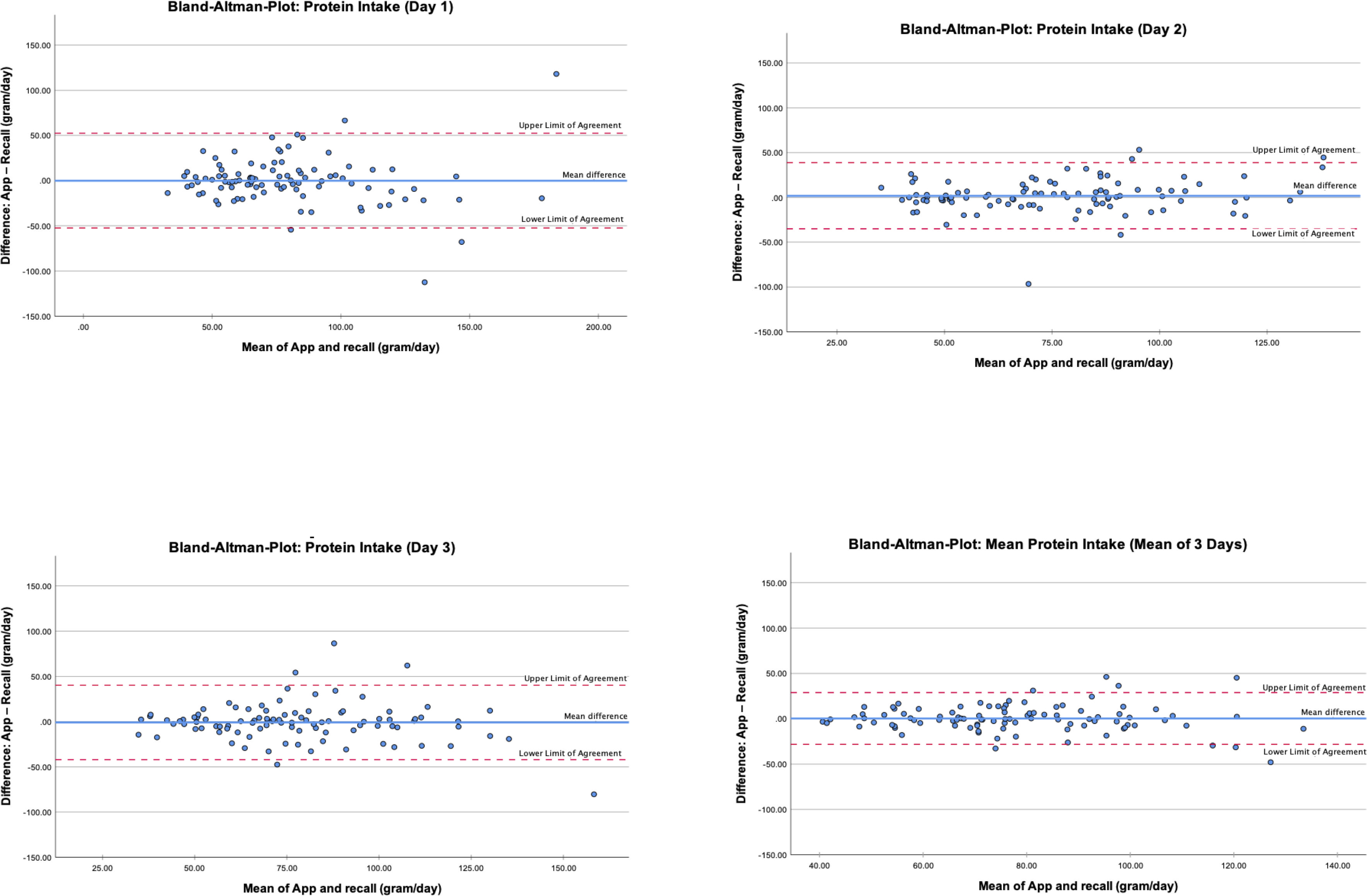

Supplement: S2 Fig — (TIF) [file pone.0337534.s002.tif]

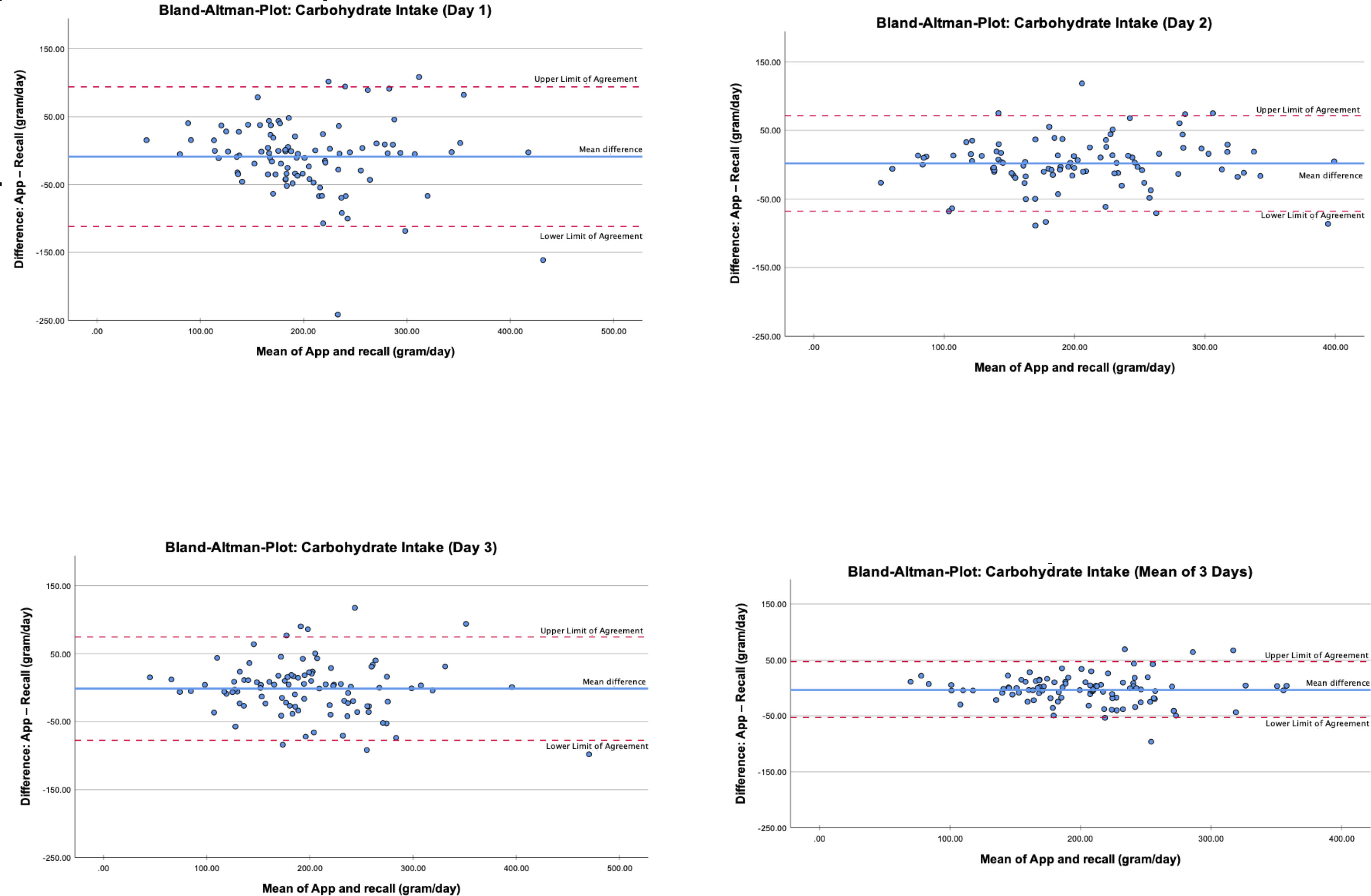

Supplement: S3 Fig — (TIF) [file pone.0337534.s003.tif]

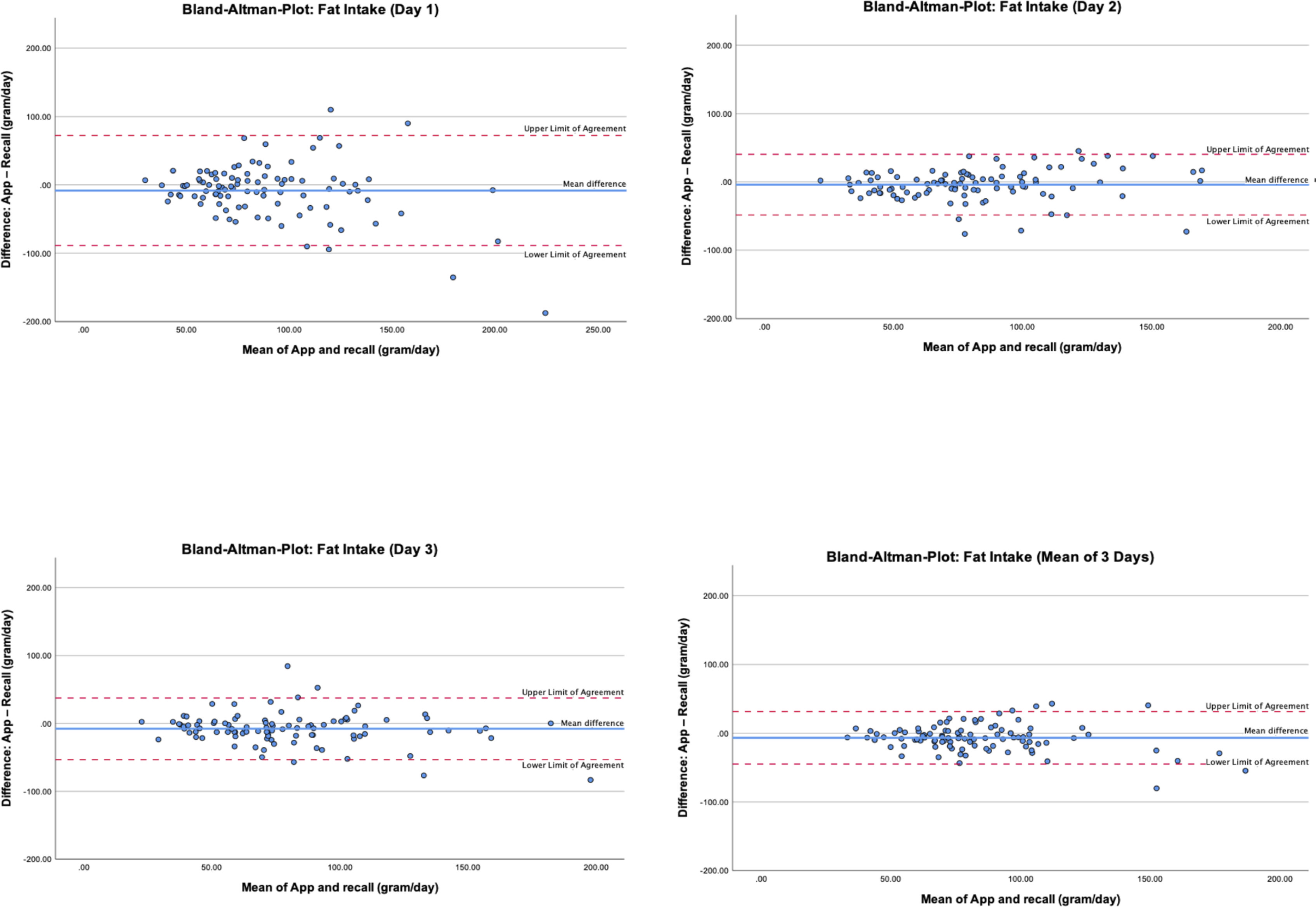

Supplement: S4 Fig — (TIF) [file pone.0337534.s004.tif]

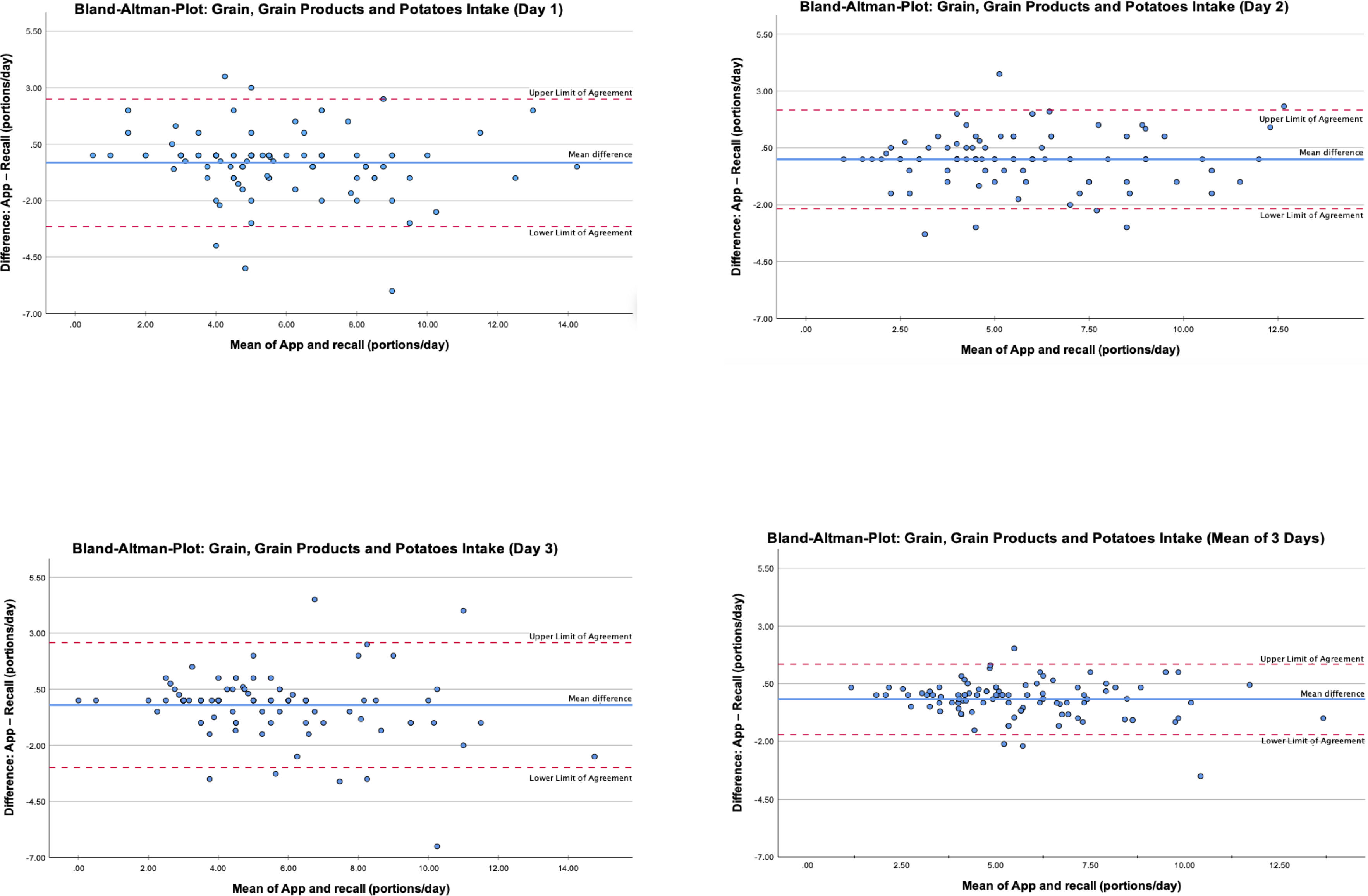

Supplement: S5 Fig — (TIF) [file pone.0337534.s005.tif]

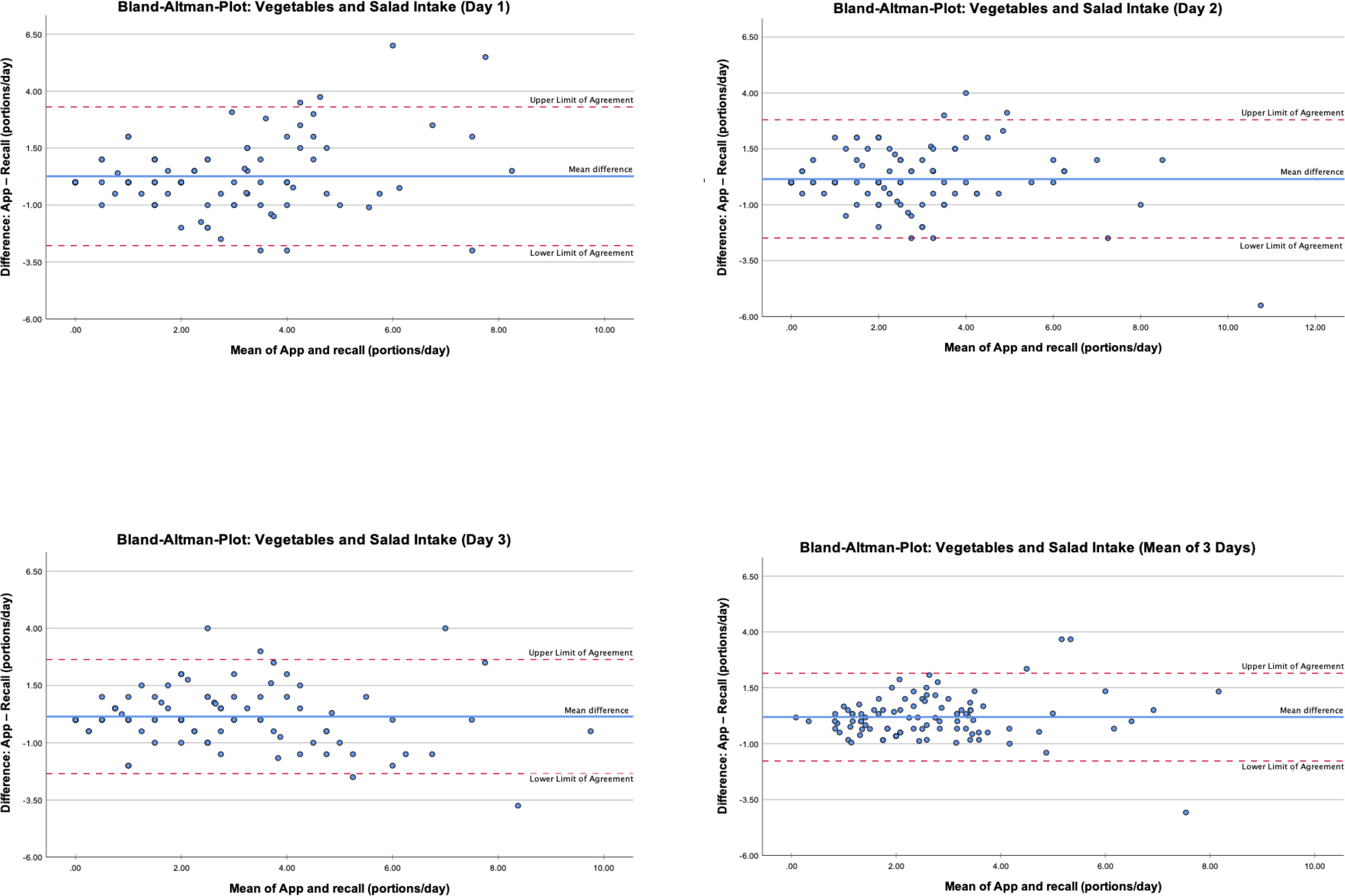

Supplement: S6 Fig — (TIF) [file pone.0337534.s006.tif]

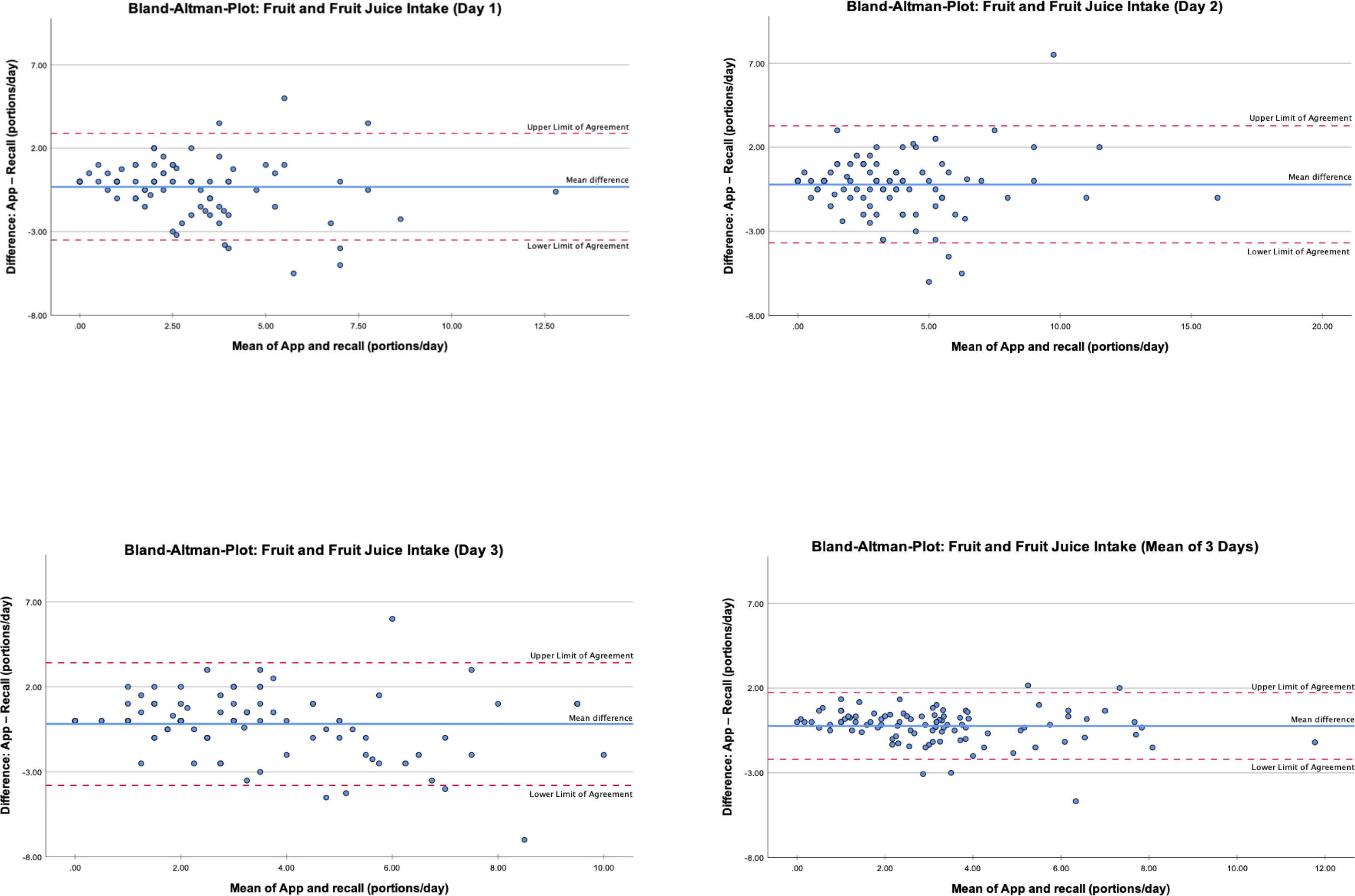

Supplement: S7 Fig — (TIF) [file pone.0337534.s007.tif]

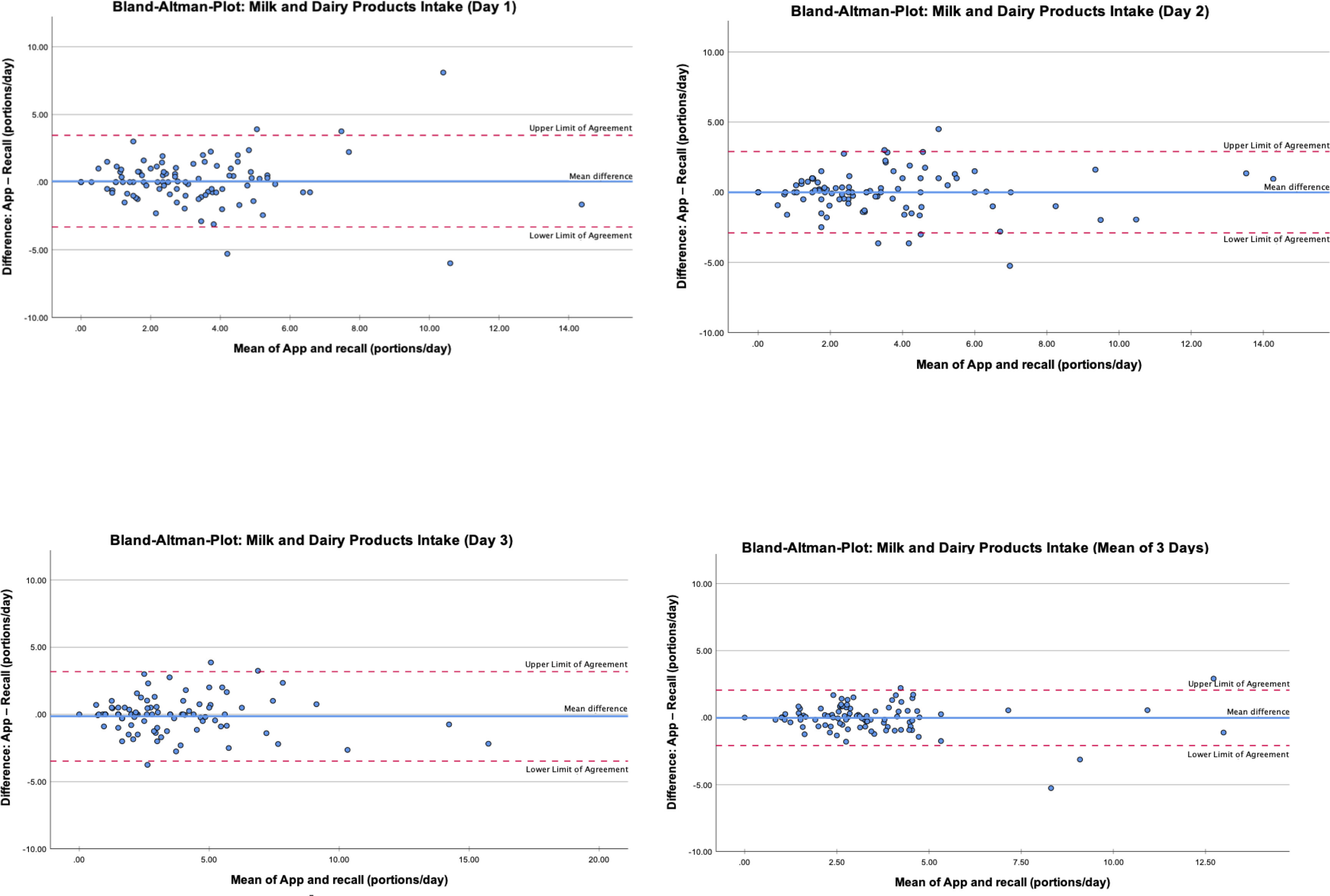

Supplement: S8 Fig — (TIF) [file pone.0337534.s008.tif]

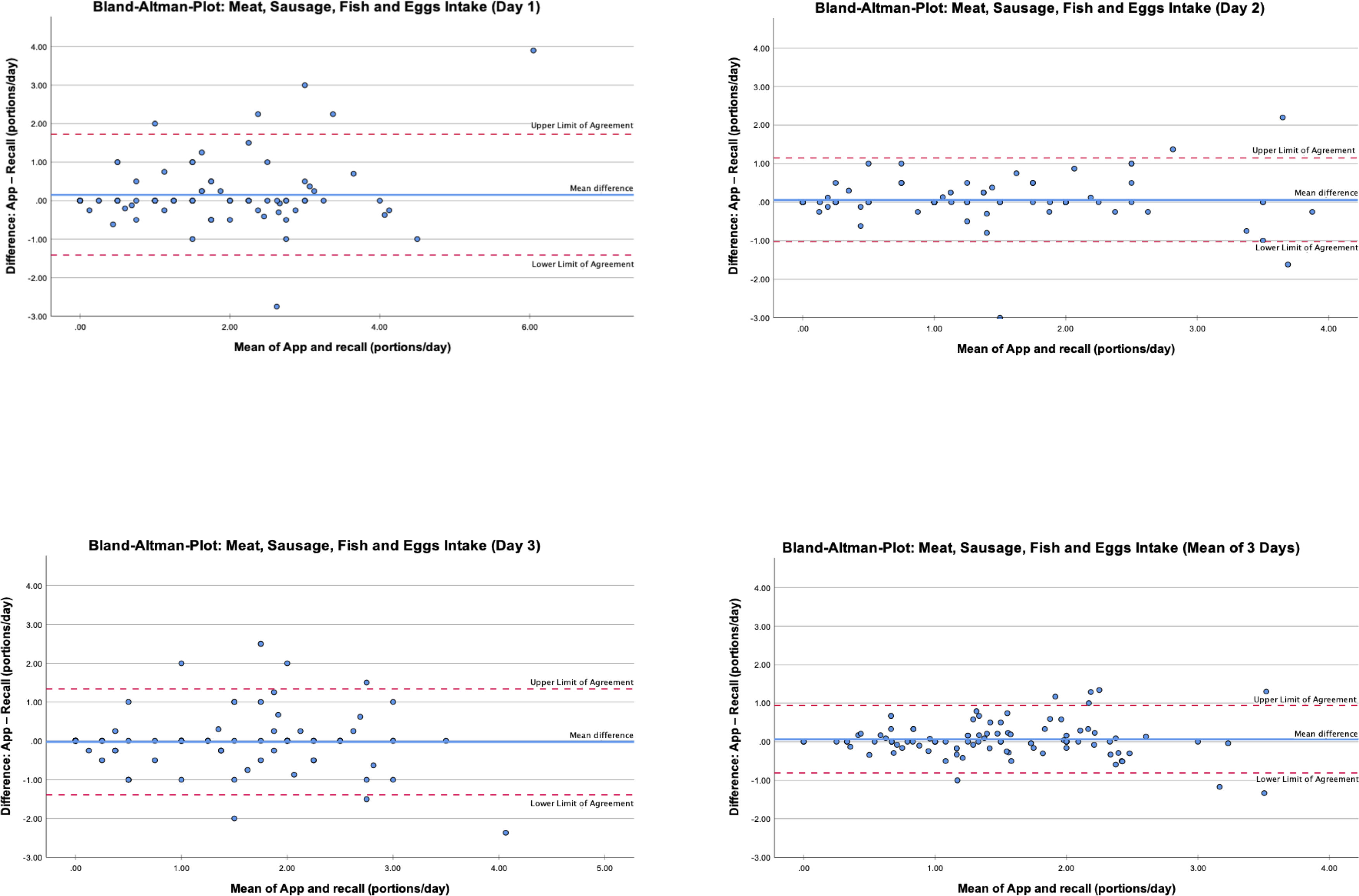

Supplement: S9 Fig — (TIF) [file pone.0337534.s009.tif]

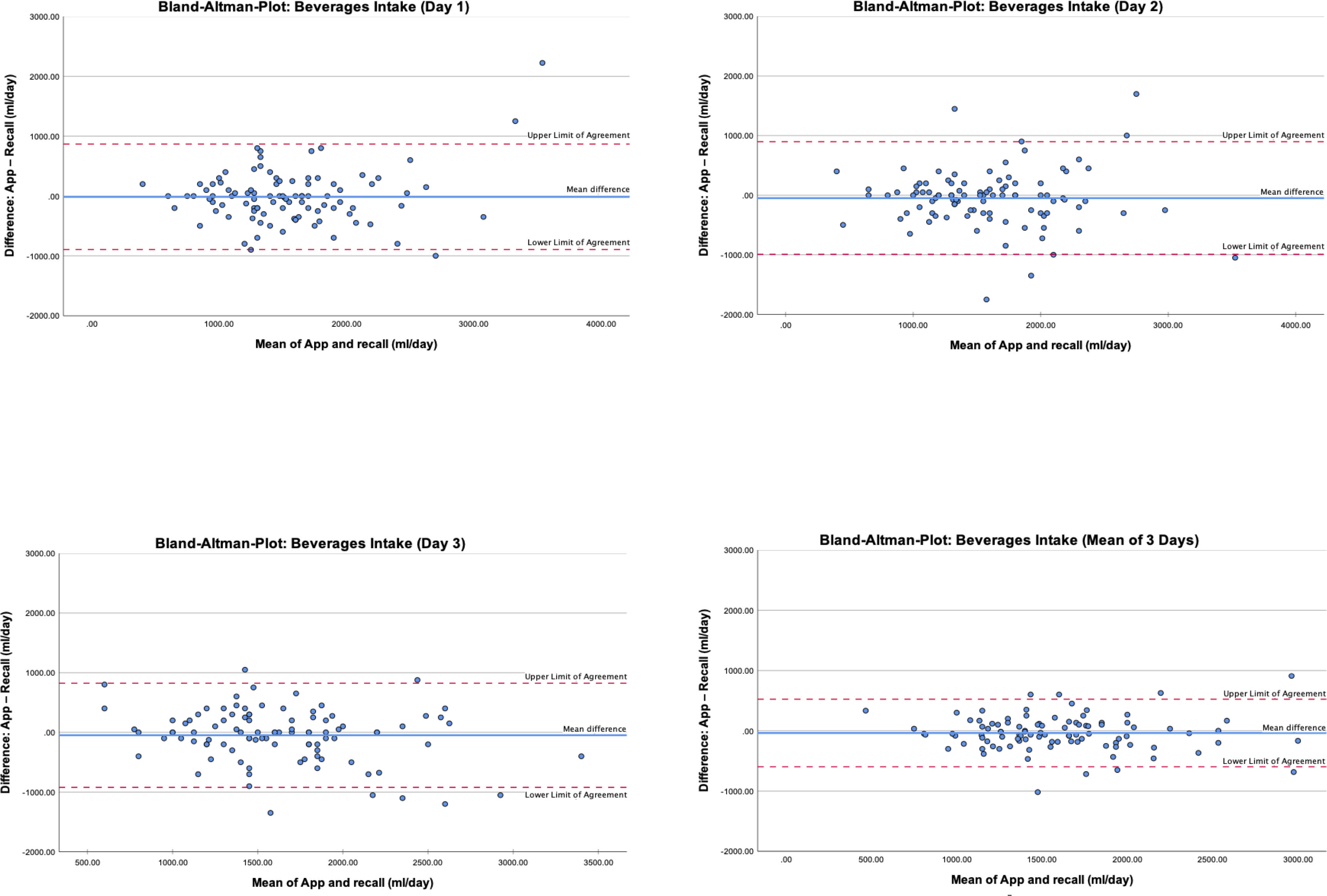

Supplement: S10 Fig — (TIF) [file pone.0337534.s010.tif]
