# Supplementary material for: Validation and evaluation of a tablet-based dietary record app for adults aged 70 and above
Source: PLoS One. 2025 Nov 25;20(11):e0337534. doi: 10.1371/journal.pone.0337534 (PMC12646446; doi:10.1371/journal.pone.0337534)
Supplement: Attachment — Submitted filename: PLOS ONE - Review app.docx [file pone.0337534.s013.docx]

PLOS ONE - Review

Manuscript Number: PONE-D-25-42816

Article Type: Research Article

**Full Title: Validation and Evaluation of a Tablet-Based Dietary Record App for Adults Aged 70**

**and Above**

**Originality and Novelty**

- The study investigates the **validation** of the NuMob-e-App dietary recording against the 24-hour recall reference among adults aged 70+, an area that is underrepresented in digital nutrition research.
- There is no evidence of the results being previously published; the manuscript contains original data from a registered study (DRKS00032600).

**Experimental, Statistical, and Analytical Standards**

- The recruitment strategy and inclusion/exclusion criteria are **clearly described**.
- The statistical analyses use accepted methods, including Two One-Sided Tests (TOST), Intraclass Correlation Coefficients (ICC), and Bland-Altman plots, with sufficiently detailed description for reproducibility.
- Data processing protocols (duplicate entry, cross-checking) are robust, minimizing error and bias.
- The sample size (n=97) is justified for validity studies in the field and covers both weekdays and weekends, reflecting typical dietary variability.
- Statistical interpretation—such as equivalence margins, Bonferroni corrections, and relative bias calculations—is technically sound and appropriately justified.

**Validity of Conclusions**

- Primary conclusions—that NuMob-e-App shows **good validity** for energy, carbohydrate, and protein intake, and some food groups, with overall agreement strongest for protein and beverages—are consistently backed by the reported ICCs, equivalence testing, and Bland-Altman analysis.
- Observed **limitations** regarding underestimation (particularly for fat), self-report biases, and possible self-selection for digital literacy are acknowledged and appropriately discussed.
- Conclusions are measured, recognizing both strengths and the boundaries of generalizability (e.g., specific to NuMob-e-App, sample may be digitally biased).

**Language and Presentation**

- The manuscript is written in **clear, standard English**, with logical structure and good flow from introduction through methods to results and discussion.
- Statistical terminology and results, such as means, standard deviations, ICCs, and Bland-Altman outcomes, are presented intelligibly for a professional readership.
- Minor typographical errors or formatting inconsistencies noted (e.g., variable font size and occasional markup artifacts) are not sufficient to detract from comprehension.

**Ethics and Research Integrity**

- The study was approved by an institutional ethics committee (Carl von Ossietzky University Oldenburg, vote 2023-172), conducted per the Declaration of Helsinki, and obtained informed consent from all participants.
- No competing interests exist; financial and funding disclosures are appropriate and transparent.
- Sensitive participant data were managed with appropriate privacy protocols and ethical statements.

**Reporting Guidelines and Data Availability**

- The reporting approach follows community standards for nutrition validation studies, including reporting on method equivalence, ICCs, and bias analysis.
- An explicit data availability statement is included, detailing that some restrictions apply due to participant consent; this aligns with current ethical guidance and journal policy.
- Registration and data repository information are referenced, and supporting figures (Bland-Altman plots) are provided.
