# Supplementary material for: Validation and evaluation of a tablet-based dietary record app for adults aged 70 and above
Source: PLoS One. 2025 Nov 25;20(11):e0337534. doi: 10.1371/journal.pone.0337534 (PMC12646446; doi:10.1371/journal.pone.0337534)
Supplement: Attachment — Submitted filename: Response to Reviewers_0.2.docx [file pone.0337534.s015.docx]

We would like to thank the reviewers for their careful reading of our manuscript and their constructive comments, which helped us to improve the paper.

We have revised the manuscript accordingly. All changes are marked in the revised version “*Revised Manuscript with track Changes*”.

**Reviewer#1**

| Reviewer Comment | Author Response |
| --- | --- |
| “Add inclusion and exclusion criteria and health state” | We thank the reviewer for this valuable suggestion. In the revised manuscript, we have added a clear description of the inclusion and exclusion criteria (see Methods section, page 5, lines 77/78).  With regard to the health state, we would like to clarify that only health conditions relevant to the safe conduct of the study (e.g., swallowing difficulties) were assessed, in order to avoid any potential risk for participants. To minimize unnecessary data collection and protect participants’ privacy, we did not record a comprehensive health status, but only information directly required for study participation. Furthermore, a complete assessment of the overall health state was not necessary, as no associations with health of the participants were analysed within the study. |
| “Good introduction but there is repetition in the idea. author can summarize all introduction in few paragraphs.” | We thank the reviewer for this helpful observation. The Introduction was revised to remove redundancies and is now re-structured. We made some changes in lines 33-35, 45, 51, 60-62 and 69. We believe this improves clarity and readability. |
| “Please re write about the app description and app services.” | Lines 117-120 and 131-136 describes the app and lines 124-129 describe the development of the app. |
| “Please summarise methods by stratified your methods you do step by step.e, .type of study, study sample, study area, study design, principles of the study ....etc, don't repeat information. It is better to re-write materials and methods.” | In preparing the manuscript, we carefully followed the section structure and subheadings required by the journal. To ensure consistency with the journal’s format, we prefer to keep the structure of Materials and Methods as following: participants and recruitment, study design, the NuMob-e App, the 24hour Recall, statistical analysis, ethical regulations. Within this structure we did some shortages (line 92, 115 and lines 152/153 and refined the MNA-SF, PAL and technical scale in lines 93-100 with the new requirements. |
| “study design is subtitle of methods” | In our manuscript, the study design is already presented as a subheading within the Methods section (Level 1 heading = Bold type, 18pt font; Level 2 Heading = Bold type, 16pt font). |
| “Better to write short description about type of statistical analysis used and its importances, using the scale of significance.” | The statistical methods used in this study (TOST, ICC, Bland-Altman analysis) are already described in the “Methods” > “Statistical analysis” section (lines167-179). Since these are established procedures, we have refrained from further explaining their general principles in order to maintain overview and have instead concentrated on the study-specific application of these methods. |
| “add descriptive table of study participants to the results” | We agree that participant characteristics are important for context. A descriptive table of study participants is already included as „*Table 1: Study characteristics of the subjects in the present study (n = 97)“*in the Results section, line 207 ff. |
| “There is mixing of the three sections(methods, results and discussion)” | After careful review, we did not identify overlaps between the Methods, Results and Discussion sections. Each section is structured according to the journal’s guidelines and presents the required content separately. |
| “Author need to write every section separated from each other including all subtitles, but links to study aim and objective.” | The sections are already separated and structured according to the journal’s author requirements. To maintain consistency with the journal’s format, we did not restructure the manuscript further. |
| “Discussion: Give brief introduction about this recent study” | In the discussion section you can find a short summary of the study aim and key findings, which serves as an introduction to the discussion of the results, please see lines 282-283. |
| “Divide your discussion into sub-title include: conclusion, recommendations, limitation of the study” | In our Discussion, we already present the results structured by statistical methods (Equivalence Testing, Intraclass Correlation Coefficients, Bland–Altman Analysis), followed by Implications and Outlook, and Qualities and Limitations. A separate Conclusion section is also included, in line with the journal’s structure. |
| “Conclusions must be shown and confirmed in the results” | Please find the conclusion at line 384 ff. |

**Reviewer#2**

| Reviewer Comment | Author Response |
| --- | --- |
| “Only minor improvements in formatting and a deeper discussion on cognitive screening and sample representativeness are warranted” | We thank the reviewer for this constructive feedback. We have revised the formatting throughout the manuscript.  Regarding cognitive screening, we clarify that participants were asked whether a diagnosis of dementia had ever been made, which served as an exclusion criterion. However, no specific cognitive test was performed, as already stated in the Discussion, and we emphasized this point further in the revised version (see lines 370-373).  With respect to sample representativeness, we acknowledge that our sample may not fully reflect the general population of older adults, since participants were volunteers in a digital nutrition study and therefore may represent a somewhat more digitally literate subgroup. This limitation was already noted in the Discussion, and we have now elaborated it more explicitly (see Discussion, lines 367-368). |

**Reviewer#3**

| Reviewer Comment | Author Response |
| --- | --- |
| “Please make sure to check your in-text citation again. In the third line of the introduction section, your in-text citation is 4??? and after that is 6 and 7??? where is 1-3 and 5 citations???” | We thank the reviewer for this careful observation! The numbering of the in-text citations was indeed inconsistent due to a formatting error with EndNote. We have carefully revised and corrected the citation order throughout the manuscript to ensure that all references now follow the correct sequence (see Introduction, lines 32-34). |
| “Is there a data related to percentage of elderly people suffering from malnutrition in Germany or any nearby country with similar setting? it is important to include such data to strengthen your idea” | We thank the reviewer for this important suggestion. Indeed, data on malnutrition prevalence among older adults in Germany was reported by Kaiser et al. (2010), showing that 22.8% suffered from malnutrition and nearly every second senior (46.2%) was at risk of malnutrition. We have included this information in the Introduction (see lines 33-35). |
| “I recommend the author (s) to seek English editing service” | We have carefully revised the manuscript to improve clarity and readability. As the manuscript was already written and reviewed in English at an advanced academic level, we believe that the current version is linguistically sound and does not require additional professional language editing. |
| “The NuMob-e-App, when was its development and release?” | Information on the development and release of the NuMob-e-App has been added in the revised manuscript (see Introduction, line 59). The App is still part of research and currently does not meet the criteria to be makted as a medical product according to MDR guidelines. |
| “In table 1, why was smoking not included?” | Smoking behaviour was not assessed in our study, as it was not relevant for the primary aim, which was to compare two dietary assessment methods. Since smoking was not part of the data collection, it cannot be added retrospectively. |
| “The quality of the figure provided is very low. maybe less than 300 dpi??? very difficult to examine it. The supplementary files figures are very clear.” | The figures have now been revised and re-submitted with improved resolution. |
